# Differential contributions of NO_3_^−^/NH_4_^+^ to nitrogen use in response to a variable inorganic nitrogen supply in plantlets of two Brassicaceae species in vitro

**DOI:** 10.1186/s13007-019-0473-1

**Published:** 2019-07-31

**Authors:** Kaiyan Zhang, Yanyou Wu, Hongtao Hang

**Affiliations:** 1 School of Karst Science, Guizhou Normal University/State Engineering Technology Institute for Karst Desertification Control, Guiyang, 550001 China; 2 State Key Laboratory of Environmental Geochemistry, Institute of Geochemistry, Chinese Academy of Sciences, No. 99 Lincheng West Road, Guanshanhu District, Guiyang, 550081 Guizhou Province People’s Republic of China; 3 CAS Center for Excellence in Quaternary Science and Global Change, Xi’an, 710061 China

**Keywords:** Ammonium, Inorganic nitrogen assimilation, Nitrate, Quantification, Stable nitrogen isotope

## Abstract

**Background:**

The primary sources of nitrogen for plants have been suggested to be nitrate (NO_3_^−^) and ammonium (NH_4_^+^). However, when both nitrate and ammonium are simultaneously available to plants, it is very difficult to differentially quantify NO_3_^−^/NH_4_^+^ utilization in culture media or soil. Consequently, the contribution of NO_3_^−^/NH_4_^+^ to total inorganic nitrogen assimilation cannot be determined.

**Results:**

We developed a method called the bidirectional stable nitrogen isotope tracer to differentially quantify the nitrate and ammonium utilization by *Orychophragmus violaceus* (*Ov*) and *Brassica napus* (*Bn*) plantlets in vitro. The utilization efficiency of nitrate was markedly lower than the utilization efficiency of ammonium for plantlets of both *Ov* and *Bn*. In both *Ov* and *Bn*, the proportion of NO_3_^−^/NH_4_^+^ utilization did not show a linear relationship with inorganic nitrogen supply. The *Ov* plantlets assimilated more nitrate than the *Bn* plantlets at the lowest inorganic nitrogen concentration.

**Conclusions:**

Quantifying the utilization of nitrate and ammonium can reveal the differences in nitrate and ammonium assimilation among plants at different inorganic nitrogen supply levels and provide an alternate way to conveniently optimize the supply of inorganic nitrogen in culture media.

**Electronic supplementary material:**

The online version of this article (10.1186/s13007-019-0473-1) contains supplementary material, which is available to authorized users.

## Background

**Fig. 1:**
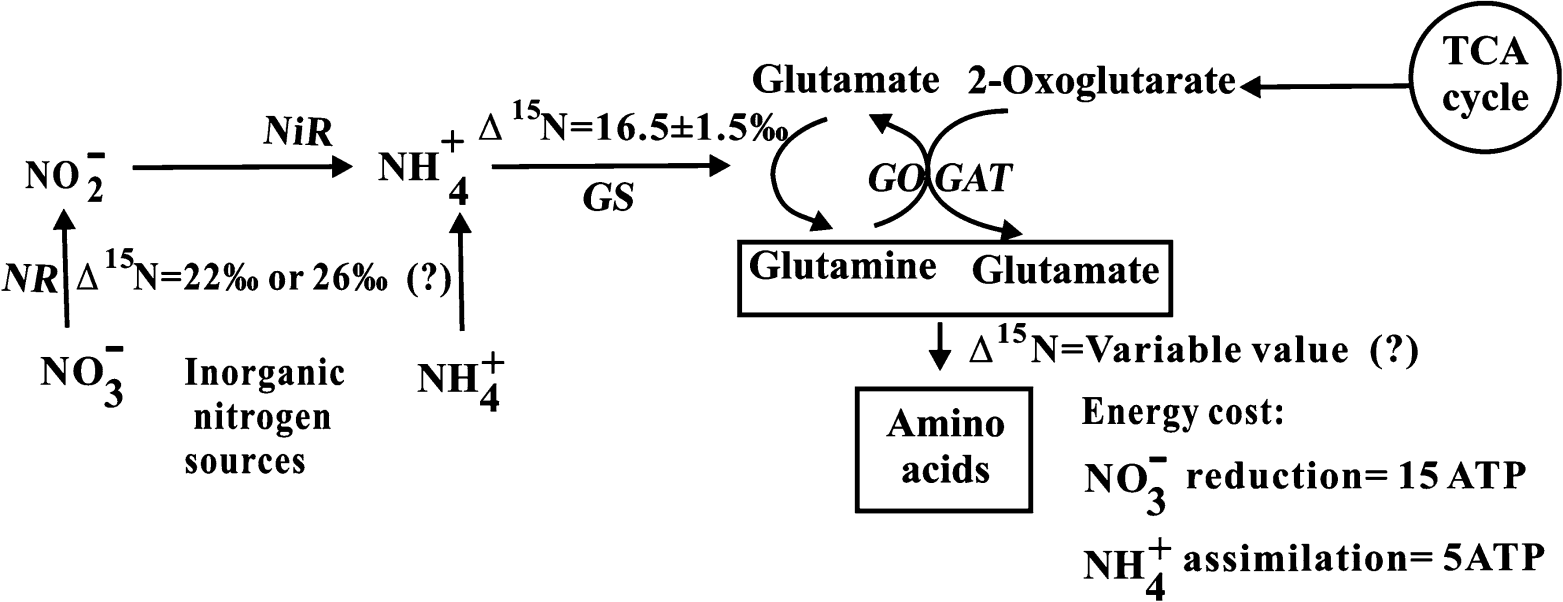
The assimilation of inorganic nitrogen in plants. The main enzymes involved are indicated in italics: *NR* nitrate reductase; *NiR* nitrite reductase; *GS* glutamine synthetase; *GOGAT* glutamate synthase. Ammonium is incorporated into organic molecules in the form of glutamine and glutamate through the combined action of the two enzymes GS and GOGAT in the plastid or chloroplast

The primary sources of nitrogen for plants generally have been suggested to be nitrate (NO_3_^−^) and ammonium (NH_4_^+^) [1–3]. The assimilation of inorganic nitrogen in plants is shown diagrammatically in Fig. 1. In aerobic soil conditions, nitrate is the major nitrogen source for most plants [4], particularly agricultural crop species. In addition to being a plant nutrient, nitrate functions in physiological processes [5]. However, the assimilation of nitrate into a plant requires energy and reductants [6]. Compared with the assimilation of ammonium, the assimilation of nitrate requires more energy [7, 8]. Moreover, nitrate assimilation leads to alkalization [9]. In contrast, the assimilation of ammonium is more advantageous because of its lower energy cost. However, many agricultural crops are sensitive to ammonium toxicity, which occurs when ammonium is the sole source of nitrogen or is present in excessive quantities (0.1–0.5 mM) [10]. In addition, ammonium assimilation leads to acidification [9], and an excess supply of ammonium causes ion disorder in plants, which is harmful to plant growth [11, 12]. In general, most plants grow well if both nitrate and ammonium are available [13].

The contents of nitrate and ammonium in agricultural soils range across three to four orders of magnitude [14]. However, the contents are even more variable in natural soils [15] and plants show variation in inorganic nitrogen utilization. Generally, the preference for a specific nitrogen form is strongly affected by the dominance of the nitrogen form in soil solution [2, 16]. Crop productivity usually has a positive relationship with nitrogen supply. However, an excess nitrogen supply will result in the waste of nitrogen fertilizers as well as environmental damage [17]. Hence, to effectively manage the inorganic nitrogen supply for plants, it is important to study the utilization proportions of nitrate and ammonium at different nitrogen levels.

The assimilation of inorganic nitrogen occurs in the roots and/or shoots of plants depending on species and available N form [18, 19]. It is difficult to study the assimilation of inorganic nitrogen in the whole plant owing to the complex patterns of transformation and distribution of nitrogen in plant organs. A simpler, more convenient approach is to study the contribution of NO_3_^−^/NH_4_^+^ utilization to total inorganic nitrogen assimilation in root-free or shoot-free plantlets. Root-free in vitro-cloned plantlets, without individual differences, are obtained by tissue culture in the presence of cytokinin and auxin concentrations that preclude root formation. These plantlets are useful for studying the contributions of nitrate and ammonium because the assimilation is restricted only to leaves.

Nitrate and ammonium are usually employed in plant tissue culture. Nitrate is a principal source of nitrogen for most plant cultures [20, 21]. For most plant cultures, the combination of nitrate and ammonium in culture media is more conducive to growth than either NO_3_^−^ or NH_4_^+^ as the sole source of nitrogen [1]. Both the growth and morphogenesis of plantlets in tissue cultures are significantly affected by the availability and forms of nitrogen [20, 21]. Nonoptimal amounts and ratios of nitrate to ammonium may result in stunted growth and physiological disorders [1, 22]. Therefore, the amount of total nitrogen and the ratio of nitrate to ammonium in culture media need to be optimized based on plant species, growth conditions, and tissue culture types [23, 24].

Murashige and Skoog (MS) [25] medium, which has a high inorganic nitrogen concentration, is widely used for most plant species. The total nitrogen concentration in the MS medium is typically 60 mM, and the ratio of nitrate to ammonium is approximately 2:1 [21]. However, for some plant cultures, the inorganic nitrogen concentration in MS medium is far above the amount required for the normal growth of plantlets in vitro, which causes much nitrogen to be wasted. In addition, in some culture media, the ratio of nitrate to ammonium is not optimal [21]. The appropriate ratio of nitrate to ammonium contributes to the optimal growth of plantlets. Therefore, it is relevant to study the proportions of assimilated nitrate and ammonium in plantlets when both nitrate and ammonium are present. However, the consumption of nitrate and ammonium and the contributions of nitrate and ammonium to total nitrogen assimilation in plantlets in vitro are difficult to precisely measure due to interference from the agar in the MS medium. Wu and Zhang [26] used near-infrared spectroscopy to determine the total nitrogen consumption in MS medium. However, they did not consider the consumption of nitrate and ammonium. At present, the optimal amount of nitrogen nutrients for plantlets in vitro is usually determined by applying a series of different concentrations of nitrate and ammonium [1, 22, 27]; this approach is very inefficient and incapable of quantifying the contributions of nitrate and ammonium. Therefore, there is a need for a high-efficiency method in which the contributions of nitrate and ammonium can be quantified to optimize the supply of inorganic nitrogen.

The nitrogen isotope composition (δ^15^N) of plants is strongly connected to the δ^15^N of the culture substrate [28, 29] and can act as an integrated measure of nitrogen uptake and assimilation [30, 31]. Hence, plant δ^15^N can be employed as an indicator of nitrogen sources [32, 33]. Moreover, the δ^15^N in plant tissue is related to the preference of a plant for an inorganic nitrogen source [2, 31]. However, nitrogen isotope fractionation occurs during nitrate assimilation by nitrate reductase (NR) or ammonium assimilation by glutamine synthetase (GS) [34] (Fig. 1). Nitrogen isotope fractionation in plants depends on the source of nitrogen [35]. The nitrogen isotope discrimination of NR approaches 22‰ [36, 37] or 26‰ [38], whereas the nitrogen isotope fractionation value of GS is 16.5 ± 1.5‰ [39]. In addition, relative to the roots, shoots are often enriched in ^15^N regardless of the inorganic nitrogen forms of NO_3_^−^ or NH_4_^+^ [40]. Therefore, nitrogen isotope fractionation should be taken into consideration when the δ^15^N values of plants are employed to study the characteristics of inorganic nitrogen assimilation.

Differential nitrogen isotope fractionation occurs during both the nitrate and ammonium assimilation processes [34]. In addition, the δ^15^N values of different amino acids distinctly differ from one another in leaves [41]. As a result, it is very difficult to simultaneously obtain the nitrogen isotope fractionation values of nitrate and ammonium during the assimilation process (Fig. 1). Usually, because of additional discrimination processes, there is a lack of accuracy and precision in differentially quantifying the contributions of nitrate and ammonium to total inorganic nitrogen assimilation when using a single isotope tracer at near-natural abundance levels. In this study, the foliar δ^15^N values of the root-free plantlets were derived from the mix of the δ^15^N values of assimilated nitrate and ammonium in leaves without interference from the assimilation of nitrate and ammonium in the roots. Considering the fact that the bidirectional stable carbon isotope tracer applied in our previous work has been successfully used to quantify the proportion of microalgal inorganic carbon utilization [42], we used two labeled stable nitrogen isotope treatments (*L*- and *H*-labeled nitrate) in this study. Moreover, the plantlets were subjected to the same culture conditions in these two labeled stable nitrogen isotope treatments. Consequently, we were able to quantify the differential contribution of nitrate/ammonium utilization to total inorganic nitrogen assimilation *via* the bidirectional stable nitrogen isotope tracer technique.

In the present study, two cruciferous plants, *Orychophragmus violaceus* (*Ov*) and *Brassica napus* (*Bn*), were employed as experimental materials. *Ov* is adapted to grow in karst regions [43], where the soil nutrient quality is poor [44] and nitrate is dominant relative to ammonium. *Bn* was used as a control. The *Orychophragmus violaceus* (*Ov*) and *Brassica napus* (*Bn*) plantlets were subjected to different inorganic nitrogen supplies. The following were our main aims: (1) to develop a method called the bidirectional stable nitrogen isotope tracer method to quantify the differential contributions of nitrate and ammonium to total inorganic nitrogen assimilation in plantlets under the presence of nitrate and ammonium in the culture media, and (2) to reveal the differences in nitrate and ammonium assimilation in each plant type among different inorganic nitrogen supply levels.

## Methods

### Plant materials and experimental treatments

The *Ov* and *Bn* plantlets in vitro were employed as explants in this experiment. Single shoots of *Ov* and *Bn* plantlets were grown in culture media with four total nitrogen concentrations. The average fresh weight (FW) per shoot was 0.09 g for the *Bn* plantlets and 0.12 g for the *Ov* plantlets. Based on the total nitrogen concentration (60 mM) in MS culture media, the total nitrogen concentrations were set as 20 mM, 40 mM, 60 mM and 80 mM in this experiment. The ratio of nitrate to ammonium within each total nitrogen concentration was 2:1. Each total nitrogen concentration contained two labeled stable nitrogen isotope treatments. The labeled treatments were separated into high (*H*) and low (*L*) natural ^15^N-abundance in NaNO_3_, with a δ^15^N of 22.67‰ in *H* and of 8.08‰ in *L*. NH_4_Cl, with a δ^15^N of − 2.64‰, was employed as the ammonium nitrogen in this experiment. Each Erlenmeyer flask (150 ml) contained 50 ml Murashige and Skoog (MS) [24] medium supplemented with 2.0 mg L^−1^ 6-benzylaminopurine (6-BA), 0.2 mg L^−1^ α-naphthylacetic acid (NAA), 3% (w/v) sucrose, and 7.5 g L^−1^ agar. The concentrations of cytokinin and auxin in this experiment precluded root formation for the plantlets in vitro. The culture media were adjusted to pH 5.8 and then autoclaved at 121 °C for 20 min. The plantlets were maintained in a growth chamber with a 12-h photoperiod (50 μmol m^−2^ s^−1^ PPFD) at 25 ± 2 °C.

### Determination of growth parameters

A 150-ml Erlenmeyer flask containing 50 ml culture substrate was weighed before cultivating each plantlet in vitro. Next, a single shoot was cultivated in the medium, and then the whole Erlenmeyer flask was weighed again. The initial fresh weight (FW) of the shoot was calculated as the difference between the first weight and second weight.

After 5 weeks of culturing, the plantlet was removed from the Erlenmeyer flask in the afternoon. The biomass of each plantlet was measured, respectively. The leaf biomass of each plantlet was also measured. The increase in biomass of each plantlet was calculated as the difference between the initial FW of the shoot and the plantlet biomass after culture for 5 weeks. In addition, the shoots of each plantlet were counted.

### Chlorophyll concentration determination

A total of 0.1 g of fresh leaf that had been triturated in a mortar with a small amount of liquid nitrogen was macerated with 15 ml 95% ethanol for 24 h at 4 °C. The absorbance of the extract at 665 and 649 nm was spectrophotometrically determined. The chlorophyll concentrations, including chlorophyll a and chlorophyll b concentrations, were determined on a fresh weight basis (mg g^−1^) and calculated using the formula of Alsaadawi [45].

### The analysis of elements and determination of δ^15^N in plantlets

At the final harvest, the leaves of each plantlet were collected and dried at 60 °C. The dried leaves were ground to a fine powder for elemental analysis and nitrogen isotope testing. The total nitrogen and carbon contents of the dried leaves were determined using an elemental analyzer (vario MACRO cube, Germany). δ^15^N was measured by a gas isotope ratio mass spectrometer (MAT-253, Germany). The δ^15^N values were calculated according to the following equation:

1 $$\updelta^{15} {\rm N}\left( \permil \right){ = (}R_{\text{sample}} /R_{\text{standard}} - 1) \times 1000$$

where *R*_sample_ refers to the nitrogen isotope ratio of the plant material, and *R*_standard_ refers to the isotope ratio of a known standard (N_2_ in air). IAEA N_1_, IAEA N_2_, and IAEA NO_3_ reference materials were used to calibrate the instrument to reach a precision of 0.2‰ [46].

### Quantification of the contributions of nitrate and ammonium to total inorganic nitrogen assimilation

The plantlets cultured with mixed-nitrogen sources assimilated the nitrate and ammonium simultaneously. Therefore, the foliar δ^15^N value of the plantlet was derived from the mix of the δ^15^N values of assimilated nitrate and ammonium. A two end-member mixing model was developed to investigate the proportions of assimilated nitrate and ammonium contributing to total inorganic nitrogen assimilation. The two end-member model was expressed as follows:

2 $$\updelta_{\text{T}} = f_{\text{A}}\updelta_{\text{A}} + f_{\text{B}}\updelta_{\text{B}} = f_{\text{A}}\updelta_{\text{A}} + \left( { 1- f_{\text{A}} } \right)\updelta_{\text{B}}$$

where δ_T_ is the foliar δ^15^N value of the plantlets cultured with mixed-nitrogen sources, which was obtained directly. δ_A_ is the δ^15^N value derived from the nitrate assimilation. δ_B_ is the δ^15^N value derived from the ammonium assimilation. *f*_A_ is the proportion of assimilated nitrate contributing to total inorganic nitrogen assimilation. *f*_B_ is the proportion of assimilated ammonium contributing to total inorganic nitrogen assimilation. Considering that many plants are sensitive to ammonium toxicity [10] and that nitrate had no adverse effects on the growth of the plants grown in media with a sole nitrogen source, we used two labeled stable nitrogen isotope treatments (*L*- and *H*-labeled nitrate) to obtain *f*_A_ and *f*_B_. In the *H* treatment, the two end-member model was expressed as follows:

3 $$\updelta_{{{\text{T}}H}} = f_{{{\text{A}}H}}\updelta_{{{\text{A}}H}} + f_{\text{B}}\updelta_{\text{B}} = f_{{{\text{A}}H}}\updelta_{{{\text{A}}H}} + \left( { 1- f_{{{\text{A}}H}} } \right)\updelta_{\text{B}}$$

In contrast, the two end-member model in the *L* treatment was expressed as follows

4 $$\updelta_{{{\text{T}}L}} = f_{{{\text{A}}L}}\updelta_{{{\text{A}}L}} + f_{\text{B}}\updelta_{\text{B}} = f_{{{\text{A}}L}}\updelta_{{{\text{A}}L}} + \left( { 1- f_{{{\text{A}}L}} } \right)\updelta_{\text{B}}$$

The plantlets were subjected to the same culture conditions in this experiment. Moreover, the culture substrate was the same in the *H* and *L* treatments. The only difference between the *H* and *L* treatments was in the δ^15^N value of the nitrate. However, the stable nitrogen isotope had no effect on the physiological processes, metabolism, growth or other parameters. Hence, there was a specific equation: *f*_A_ = *f*_A*H*_ = *f*_A*L*_, 1 − *f*_A*H*_ = 1 − *f*_A*L*_, which evolved to a simplified equation that was written as follows:

5 $$f_{\text{A}} = \left( {\updelta_{{{\text{T}}H}} -\updelta_{{{\text{T}}L}} } \right)/ \, \left( {\updelta_{{{\text{A}}H}} -\updelta_{{{\text{A}}L}} } \right)$$

The standard deviation (SD) of *f*_A_ was achieved by the error propagation formula.

When the plantlets were cultured in the medium with mixed-nitrogen sources, it would have been difficult to directly obtain δ_A*L*_ and δ_A*H*_, which were involved in the nitrogen isotope discrimination in nitrate assimilation and the exchange of unassimilated nitrate between the shoot and the substrate during the whole culture period. Therefore, δ_A*L*_ and δ_A*H*_ changed over time in this experiment. However, we were able to obtain δ_A*L*_ and δ_A*H*_ when the plantlets were grown in the culture medium in which the nitrate was the sole nitrogen source.

The δ_A*L*_ and δ_A*H*_ in NO_3_^−^-fed plantlets could be affected by unassimilated nitrate. Nevertheless, their study found that the storage pool of nitrate in leaves of tomato and tobacco plants were replenished in the dark and became depleted in the light, and the nitrate concentration in tomato and tobacco leaves reached a low level in the afternoon [47, 48]. Therefore, after the plantlets had been cultured for 5 weeks and harvested in the afternoon, the amount of unassimilated nitrate in leaves of plantlets would be very small in comparison with the amount of assimilated nitrate. In addition, the foliar δ^15^N value of plantlets did not vary significantly among nitrate concentrations ranging from 10 to 40 mM [49], which suggested that the effect of unassimilated nitrate in leaves on the foliar δ^15^N value could be ignored. Accordingly, the δ_A*L*_ and δ_A*H*_ of plantlets cultured in the medium with mixed-nitrogen sources could be replaced by the δ_A*L*_ and δ_A*H*_ in NO_3_^−^-fed plantlets.

In this study, the foliar δ^15^N values of NO_3_^−^-fed plantlets that had been cultured for 5 weeks could be regarded as the δ^15^N values (δ_A*L*_ or δ_A*H*_) of plantlets cultured in the medium with mixed-nitrogen sources. Zhang and Wu [49] found that the foliar δ^15^N values of plantlets did not vary significantly among nitrate concentrations in the culture medium ranging from 10 to 40 mM. Sodium nitrate, the δ^15^N value of which was 8.08‰, was employed as the sole nitrogen source in their experiment. Accordingly, in our experiment, sodium nitrate with a δ^15^N of 22.67‰ was used as the sole nitrogen source, and three nitrate supply levels (10, 20, and 40 mM) were applied. The plantlets were grown in the above-described culture medium. Similar to Zhang and Wu [49], we found that the foliar δ^15^N values of the plantlets did not vary significantly among nitrate concentrations ranging from 10 to 40 mM. Therefore, the average foliar δ^15^N value in NO_3_^−^-fed plantlets at the three nitrate supply levels (10, 20, and 40 mM) was approximately equal to the δ^15^N value (δ_A*L*_ or δ_A*H*_) of plantlets cultured in the medium with mixed-nitrogen sources. As a result, we were able to obtain δ_A*L*_ and δ_A*H*_. δ_A*L*_ was 5.71 ± 0.51‰ (n = 9) for the *Ov* plantlets and 3.17 ± 0.35‰ (n = 9) for the *Bn* plantlets [49], and δ_A*H*_ was 17.02 ± 0.68‰ (n = 9) for the *Ov* plantlets and 15.19 ± 0.86‰ (n = 9) for the *Bn* plantlets. After determining δ_T*H*_, δ_T*L*_, δ_A*H*_ and δ_A*L*_, we were able to calculate *f*_A_ and *f*_B_.

### The contribution of NO_3_^−^/NH_4_^+^ utilization to the amount of nitrogen in chlorophyll a

The amount of chlorophyll a (m_chla_) was calculated using the following equation:

6 $${\text{m}}_{\text{chla}} = {\text{FW}} \times {\text{c}}_{\text{chla}}$$

where the FW is the fresh weight of all leaves in each plantlet, and c_chla_ is the concentration of chlorophyll a (Chla).

Because one mole Chla molecule contains four moles N, the amount of nitrogen in Chla (Chla-N) is 6.28% of the m_chla_. Accordingly, the amount of Chla-N (m_chla-N_) derived from the assimilated nitrate and ammonium was calculated by the following equations:

7 $${\text{m}}_{\text{chla}\,{-}\,{\text{N(nitrate)}}} = { 0} . 0 6 2 8\times {\text{m}}_{\text{chla}} \times f_{\text{A}}$$

8 $${\text{m}}_{\text{chla}\,{-}\,{\text{N(ammonium)}}} = { 0} . 0 6 2 8\times {\text{m}}_{\text{chla}} \times f_{\text{B}}$$

where m_chla–N(nitrate)_ is the amount of Chla-N derived from nitrate assimilation, and m_chla−N(ammonium)_ is the amount of Chla-N derived from ammonium assimilation. The standard deviation (SD) of m_chla–N(nitrate)_ and m_chla−N(ammonium)_ was calculated by the error propagation formula.

### Statistical analysis

The data were subjected to an analysis of variance (ANOVA). The means of the different groups were compared *via* Tukey’s test (*p* < 0.05). The data are shown as the mean ± standard deviation (SD).

## Results

### Growth

The effect of inorganic nitrogen concentration on growth differed between the species (Table 1). The biomass increase of the *Ov* plantlets did not markedly vary over total nitrogen supply levels from 20 to 80 mM. However, increasing the inorganic nitrogen supply promoted the growth of the *Bn* plantlets. The *Ov* plantlets had a greater biomass than the *Bn* plantlets at the lowest total nitrogen supply.

**Table 1:** The growth parameters of the *Ov* and *Bn* plantlets cultured under different inorganic nitrogen concentrations

Parameters	Plant species	Inorganic nitrogen concentration (mM)			
		20	40	60	80
Increased biomass (g)	*Ov*	3.45 ± 0.07a	3.45 ± 0.59a	2.85 ± 0.42a	3.11 ± 0.54a
	*Bn*	2.41 ± 0.41ab	2.36 ± 0.04b	2.81 ± 0.16ab	3.06 ± 0.28a
Number of shoots	*Ov*	8.0 ± 1.0ab	8.7 ± 0.6a	6.0 ± 1.0bc	5.0 ± 1.0c
	*Bn*	5.7 ± 0.6ab	5.0 ± 1.0b	5.7 ± 1.2ab	7.3 ± 0.6a

*Ov Orychophragmus violaceus*, *Bn Brassica napus*. The ratio of nitrate to ammonium within each inorganic nitrogen concentration was 2:1. Each value represents the mean ± SD (n = 3). Values signed with the same letter in each line are not significantly different by Tukey’s test (p > 0.05)

With respect to the proliferation of shoots, the *Ov* and *Bn* plantlets showed different responses to increasing inorganic nitrogen concentrations. The number of shoots of *Ov* plantlets declined significantly when the total nitrogen supply increased from 40 to 60 mM. In contrast, the number of shoots of *Bn* plantlets did not markedly vary among total nitrogen concentrations ranging from 20 to 60 mM. Generally, both the *Ov* and *Bn* plantlets had good performance with respect to shoot proliferation under all treatments (Table 1).

### Chlorophyll concentrations

The chlorophyll concentrations of the plantlets of both *Ov* and *Bn* were significantly affected by the total nitrogen supply. Increasing the supply of inorganic nitrogen promoted the biosynthesis of chlorophyll in both *Ov* and *Bn* plantlets. The *Ov* plantlets synthesized more chlorophyll than the *Bn* plantlets under each treatment (Table 2).

**Table 2:** The chlorophyll concentration of the *Ov* and *Bn* plantlets cultured under different inorganic nitrogen concentrations

Parameters	Plant species	Inorganic nitrogen concentration (mM)			
		20	40	60	80
chl a (mg/g)	*Ov*	0.59 ± 0.04a	0.70 ± 0.10a	0.75 ± 0.09a	0.79 ± 0.10a
	*Bn*	0.50 ± 0.05b	0.53 ± 0.06b	0.70 ± 0.03a	0.78 ± 0.08a
chl b (mg/g)	*Ov*	0.30 ± 0.04b	0.35 ± 0.02ab	0.37 ± 0.05ab	0.40 ± 0.03a
	*Bn*	0.17 ± 0.03b	0.19 ± 0.02b	0.22 ± 0.03ab	0.28 ± 0.03a
chl a + b (mg/g)	*Ov*	0.89 ± 0.06b	1.05 ± 0.09ab	1.12 ± 0.13ab	1.19 ± 0.11a
	*Bn*	0.67 ± 0.07c	0.72 ± 0.08bc	0.92 ± 0.06ab	1.05 ± 0.11a

*Ov*-*Orychophragmus violaceus*, *Bn*-*Brassica napus*. The ratio of nitrate to ammonium within each inorganic nitrogen concentration was 2:1. Each value represents the mean ± SD (n = 3). Values signed with the same letter in each line are not significantly different by Tukey’s test (p > 0.05)

### Elemental analysis of the *Ov* and *Bn* plantlets

**Fig. 2:**
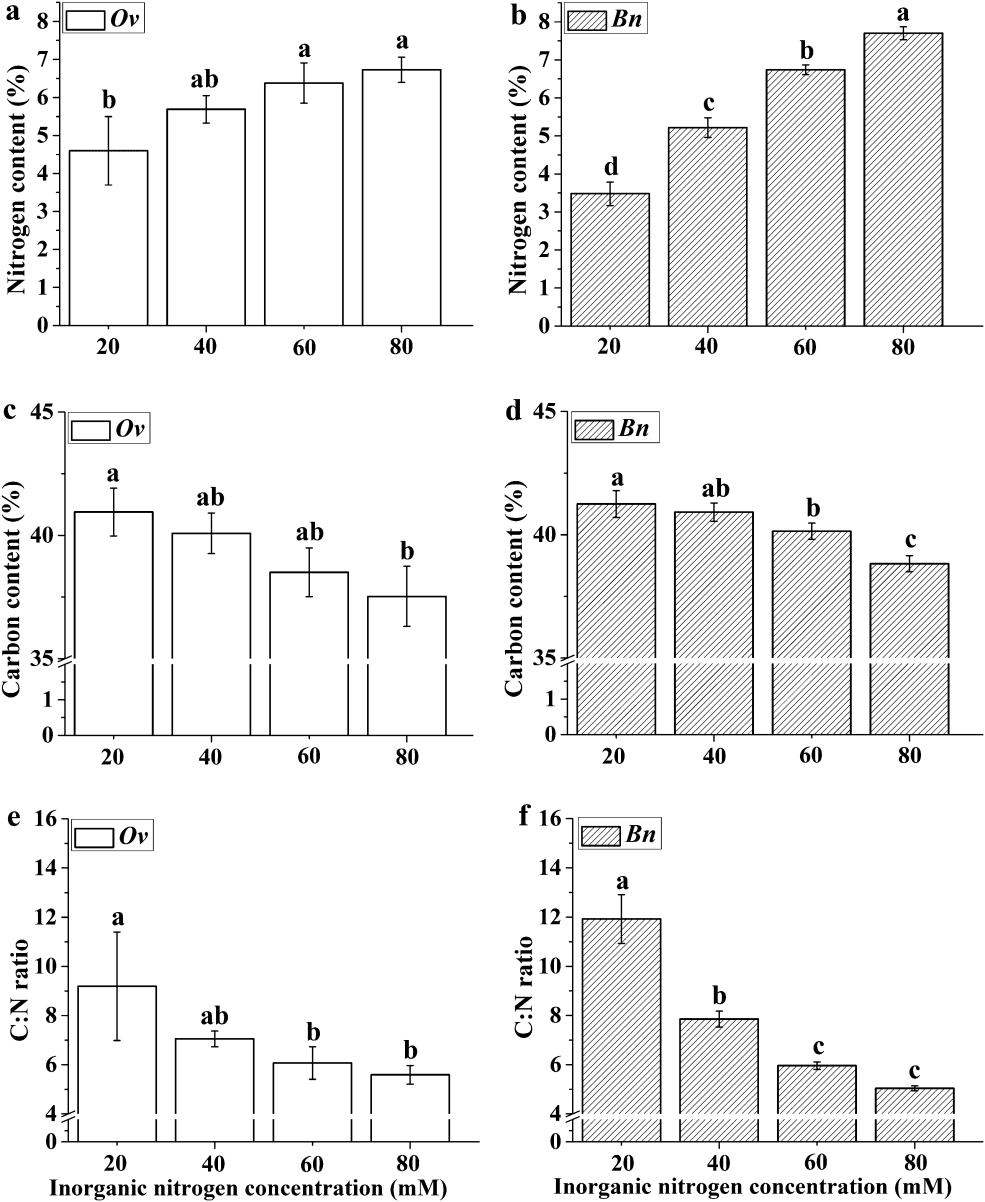
Nitrogen content (**a**, **b**), carbon content (**c**, **d**) and C:N ratio (**e**, **f**) of the *Ov* and *Bn* plantlets cultured under different inorganic nitrogen concentrations. *Ov Orychophragmus violaceus*, *Bn Brassica napus*. The ratio of nitrate to ammonium within each inorganic nitrogen concentration was 2:1. The nitrogen and carbon content was expressed as a percent of foliar dry weight, respectively. The mean ± SD (n = 3) followed by different letters in the same plant species differ significantly (Tukey’s test, p < 0.05)

Increasing the inorganic nitrogen supply promoted nitrogen accumulation in plantlet leaves for both *Ov* and *Bn*. The leaf nitrogen content of *Bn* plantlets increased significantly with increasing inorganic nitrogen supply. However, the leaf nitrogen content of *Ov* plantlets did not significantly increase from 40 to 80 mM of total nitrogen supply. In addition, the *Ov* plantlets accumulated more nitrogen than *Bn* plantlets at the lowest level of inorganic nitrogen supply. In contrast to the leaf nitrogen content, the leaf carbon content of plantlets of both *Ov* and *Bn* gradually declined with increasing inorganic nitrogen concentration. Accordingly, the C:N ratio of the *Ov* and *Bn* plantlets declined with increasing inorganic nitrogen supply (Fig. 2).

### Foliar nitrogen isotope ratio

**Fig. 3:**
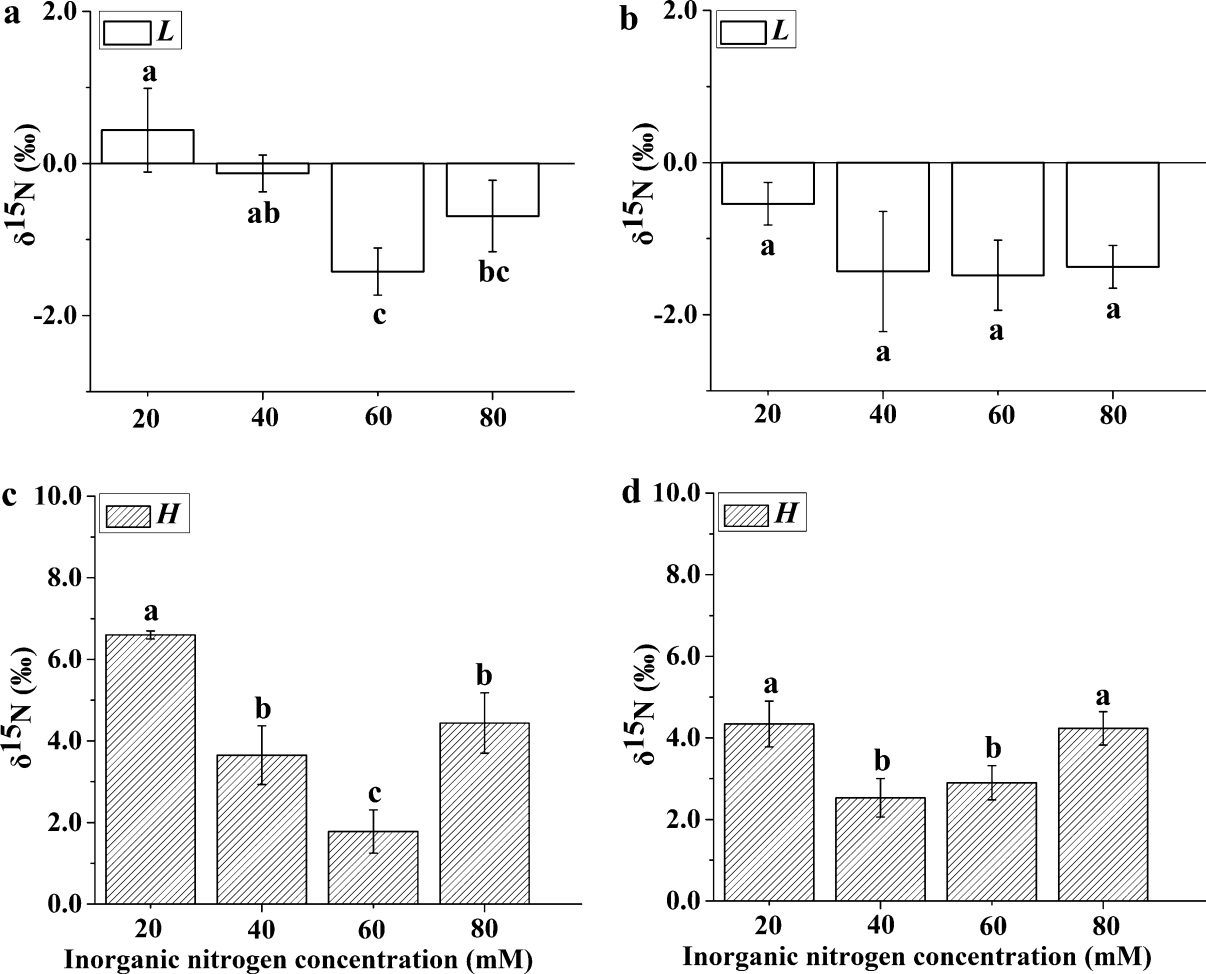
The foliar δ^15^N values of the *Ov* (**a**, **c**)and *Bn* (**b**, **d**) plantlets cultured under different inorganic nitrogen concentrations. *Ov Orychophragmus violaceus*, *Bn Brassica napus*. The ratio of nitrate to ammonium within each inorganic nitrogen concentration was 2:1. The mean ± SD (n = 3) followed by different letters in the same legend differ significantly (Tukey’s test, p < 0.05)

The δ^15^N values of plantlets of both *Ov* and *Bn* cultured in the *H* and *L* treatments were very different at all levels of inorganic nitrogen supply. The δ^15^N values of the *Ov* and *Bn* plantlets in each treatment were different from those of the substrate. The δ^15^N values of the plantlets were higher in the *H* treatment than in the *L* treatment for both *Ov* and *Bn*. The δ^15^N values of the *Ov* and *Bn* plantlets first decreased and then increased with increasing inorganic nitrogen concentration. In both the *L* and *H* treatments, the maximum and minimum δ^15^N values of *Ov* plantlets occurred at 20 mM and 60 mM inorganic nitrogen, respectively. The δ^15^N value of the *Ov* plantlets was significantly affected by inorganic nitrogen concentration in both the *H* and *L* treatments. However, the δ^15^N value of the *Bn* plantlets did not change significantly with increasing inorganic nitrogen concentration in the *L* treatment (Fig. 3).

### The contribution of nitrate/ammonium to total inorganic nitrogen assimilation

**Fig. 4:**
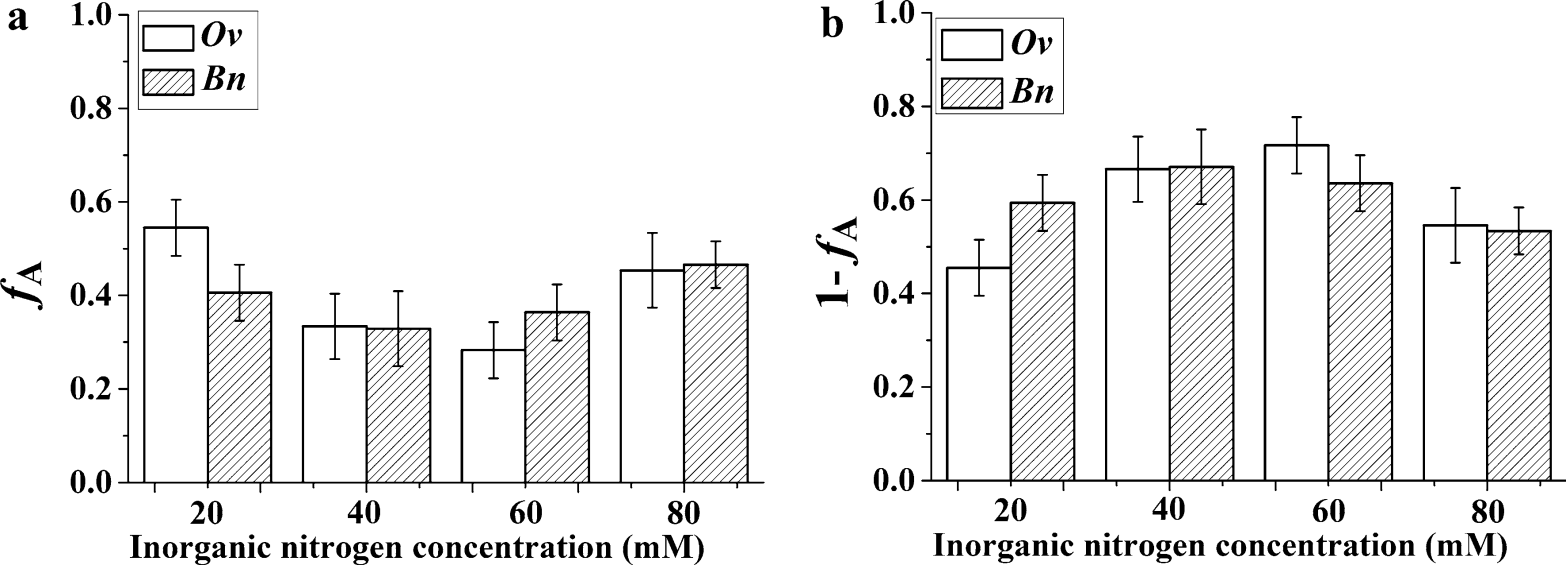
The contribution of the nitrate (**a**) and ammonium utilization (**b**) to total inorganic nitrogen assimilation for the *Ov* and *Bn* plantlets cultured under different inorganic nitrogen concentrations. *Ov Orychophragmus violaceus*, *Bn Brassica napus*. The ratio of nitrate to ammonium within each inorganic nitrogen concentration was 2:1. The error bars was the result which was calculated by the error propagation formula

The inorganic nitrogen concentration had a significant effect on the contributions of assimilated nitrate and ammonium to total inorganic nitrogen assimilation for both the *Ov* plantlets and the *Bn* plantlets. The contribution of nitrate utilization to total inorganic nitrogen assimilation was higher at 20 mM and 80 mM total nitrogen than at the other concentrations for both the *Ov* and *Bn* plantlets. The contribution of nitrate utilization in the *Ov* plantlets was much higher than that in the *Bn* plantlets at 20 mM total nitrogen. However, the ammonium utilization was the major contributor to plant nitrogen for the *Ov* and *Bn* plantlets at 40 mM and 60 mM total nitrogen (Fig. 4). In general, ammonium was the primary source of nitrogen that was assimilated by the *Ov* and *Bn* plantlets at a sufficient nitrogen supply.

### The contributions of assimilated nitrate/ammonium to the amount of nitrogen in Chla

**Fig. 5:**
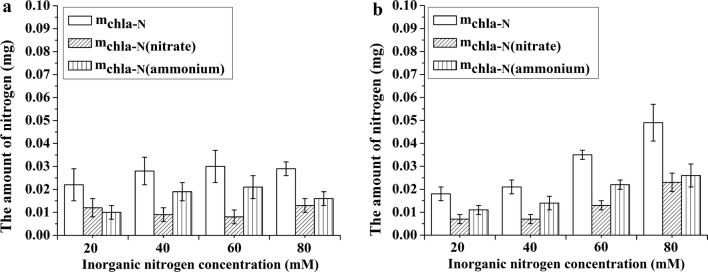
The amount of nitrogen in Chla (m_chla-N_), the amount of Chla-N (m_chla-N(nitrate)_) derived from the nitrate assimilation, and the amount of Chla-N (m_chla-N(ammonium)_) derived from the ammonium assimilation of the *Ov* (**a**) and *Bn* (**b**) plantlets. Note: The ratio of nitrate to ammonium within each inorganic nitrogen concentration was 2:1. The error bars of m_chla-N(nitrate)_ and m_chla-N(ammonium)_ were calculated by the error propagation formula

The amount of Chla-N in response to increasing inorganic nitrogen supply differed between the *Ov* and *Bn* plantlets. The amount of Chla-N in the *Bn* plantlets increased linearly with increasing inorganic nitrogen supply, whereas that in the *Ov* plantlets first increased and then remained approximately constant (Fig. 5). Moreover, the maximum amount of Chla-N in the *Bn* plantlets was markedly higher than that in the *Ov* plantlets. The amount of Chla-N in the *Bn* plantlets derived from nitrate and ammonium utilization increased continuously with increasing inorganic nitrogen supply. The amount of Chla-N in the *Ov* plantlets derived from nitrate utilization declined slowly with increasing inorganic nitrogen supply, except at the maximum inorganic nitrogen concentration. The amount of Chla-N in the *Ov* plantlets derived from ammonium utilization initially increased with increasing inorganic nitrogen supply but then decreased at the maximum inorganic nitrogen concentration (Fig. 5).

## Discussion

### The method of quantifying the contribution of assimilated nitrate/ammonium to total inorganic nitrogen assimilation

The nitrogen form had a pronounced effect on the δ^15^N values of the plants. Kalcsits et al [31] found that the δ^15^N value in NO_3_^−^-fed plants was very different from that in NH_4_^+^-fed plants. Otherwise, both the efflux of nitrate and ammonium to the external media [50] and the assimilation of nitrate and ammonium could affect the nitrogen isotope discrimination [51]. In this study, the δ^15^N values of the plantlets of both *Ov* and *Bn* showed large differences between the *L*- and *H*-labeled treatments (Fig. 3). The foliar δ^15^N value was derived from the mix of the δ^15^N values of assimilated nitrate and ammonium in the leaves because no root formation occurred in the *Ov* and *Bn* plantlets in this experiment. The δ^15^N values of both the *Ov* and *Bn* plantlets in each treatment were different from those of the substrate, which suggested that nitrogen isotope fractionation occurred during the assimilation of the inorganic nitrogen in both the *Ov* and *Bn* plantlets. Zhang and Wu [49] found that nitrogen isotope fractionation occurred during nitrate assimilation, as evidenced by the lower δ^15^N in the NO_3_^−^-fed plantlets than in the substrate in their experiment. Furthermore, their study suggested that nitrogen isotope fractionation also occurred during ammonium assimilation [31, 52, 53]. Hence, it would not have been possible for us to distinguish the differential contributions of NO_3_^−^/NH_4_^+^ to nitrogen use from the foliar δ^15^N of plants grown in a mixed-nitrogen source with a single isotope tracer at near-natural abundance levels.

The δ^15^N values of plantlets of both *Ov* and *Bn* at the four inorganic nitrogen levels suggested that the contributions of nitrate and ammonium differed from each other within each inorganic nitrogen treatment. However, the contributions of assimilated nitrate and ammonium were unlikely to be obtained from the δ^15^N values in the *L*- or *H*-labeled treatment. In addition, increasing the inorganic nitrogen supply significantly improved foliar nitrogen content in both the *Ov* and *Bn* plantlets. However, it is unclear how much nitrate/ammonium contributes to inorganic nitrogen assimilation.

The δ^15^N in plants has a positive relationship with the δ^15^N of the growth substrate [28]. Therefore, when the δ^15^N values of nitrate and ammonium were different and the nitrogen isotope fractionation values of assimilated nitrate and ammonium were known during nitrogen assimilation, we were able to quantify the contribution of assimilated nitrate/ammonium to total inorganic nitrogen assimilation with the δ^15^N values of the root-free plantlets. However, it was very difficult to simultaneously obtain the nitrogen isotope fractionation value of the plantlets, which was derived from the nitrate and ammonium assimilation, when the nitrate and ammonium were present in the culture medium.

In this study, it was unnecessary to simultaneously obtain the nitrogen isotope fractionation values of the plantlets, which were derived from the nitrate and ammonium assimilation, when the two labeled stable nitrogen isotope treatments were used. As shown in Eq. (5), the contribution of nitrate assimilation depended only on δ_T*H*_, δ_T*L*_, δ_A*L*_ and δ_A*H*_. δ_T*H*_ and δ_T*L*_ were the foliar δ^15^N values of the plantlets grown in the mixed-nitrogen source and could be obtained directly. δ_A*L*_ and δ_A*H*_ could be replaced by the foliar δ^15^N values of the plantlets grown in the corresponding culture medium in which nitrate was the sole nitrogen source. As a result, we were able to successfully quantify the contribution of assimilated nitrate/ammonium to total inorganic nitrogen assimilation *via* the bidirectional stable nitrogen isotope tracer technique.

### The contribution of NO_3_^−^/NH_4_^+^ in response to a variable inorganic nitrogen supply

The contribution of assimilated nitrate/ammonium to total inorganic nitrogen assimilation was affected by the inorganic nitrogen concentration (Fig. 4), in which the ratio of nitrate to ammonium was 2:1. Nitrate was the main source of nitrogen assimilated by the *Ov* plantlets at 20 mM total nitrogen. Ammonium was the major source of nitrogen assimilated by the *Bn* plantlets at all inorganic nitrogen concentrations. Our results suggested that the *Ov* plantlets assimilated more nitrate than the *Bn* plantlets at 20 mM total nitrogen; i.e., the nitrate assimilation ability of the *Ov* plantlets was higher than that of the *Bn* plantlets at 20 mM total nitrogen. Zhang and Wu [49] formed the same conclusion based on the nitrogen isotope fractionation of nitrate for *Ov* and *Bn* plantlets. Considering the low inorganic nitrogen concentration in karst regions, where nitrate is more abundant than ammonium [54], the *Ov* plantlets, with their strong ability to assimilate nitrate at low nitrate concentrations, would have an advantage in acquiring available nitrogen to survive in karst regions.

With increasing inorganic nitrogen concentration, the proportion of assimilated nitrate was low for both the *Ov* plantlets and the *Bn* plantlets. At 40 mM and 60 mM total nitrogen, the foliar nitrogen content of the plantlets of both *Ov* and *Bn* was mainly derived from ammonium assimilation. The difference between nitrate assimilation and ammonium assimilation might have been related to differences in energy cost. Ammonium assimilation uses less energy than does nitrate assimilation [7]. Therefore, ammonium assimilation was predominant for both *Ov* and *Bn*. However, the proportion of assimilated ammonium was not highest at the maximum inorganic nitrogen concentration in the culture medium, which might be attributed to the futile cycling of ammonium nutrition due to high ammonium concentration [55, 56]. The relationship between the total inorganic nitrogen supply and biomass suggests that the maximum level of inorganic supply was not optimal for either the *Ov* plantlets or the *Bn* plantlets. Moreover, the maximum level of inorganic supply represented a waste of nitrogen fertilizer.

The nitrogen accumulation in leaves could indicate the nitrogen acquisition capacity among plants at different inorganic nitrogen supply levels. Among the nitrogen-containing substances in the plant, the Chla is easy to measure. Therefore, the amount of Chla-N was presented as an example to represent the nitrogen accumulation in leaves. In this study, we found that the inorganic nitrogen supply affected the amount of Chla-N of plantlets for both *Ov* and *Bn*. The amount of Chla-N in the *Bn* plantlets increased continuously with increasing inorganic nitrogen supply. However, the amount of Chla-N in the *Ov* plantlets tended to remain constant at 40 mM total nitrogen concentration (Fig. 5). The above results suggested that the ability to acquire inorganic nitrogen was different between the *Ov* plantlets and the *Bn* plantlets. With increasing inorganic nitrogen supply, the nitrogen accumulation in the *Bn* plantlets, which was derived from the assimilation of nitrate and ammonium, increased accordingly. The nitrogen accumulation in the *Bn* plantlets depended on the supply of nitrate and ammonium. However, in the *Ov* plantlets, when the supply of nitrate and ammonium exceeded a certain level, the nitrogen accumulation ceased to increase with increasing inorganic nitrogen supply. Ammonium contributed most of the Chla-N for the *Ov* and *Bn* plantlets, which might reflect the fact that ammonium assimilation requires less energy than does nitrate assimilation [7]. Because the amount of Chla-N in the *Ov* plantlets did not markedly change between 40 mM to 80 mM inorganic nitrogen and because the proportion of ammonium assimilation declined at 80 mM inorganic nitrogen, the amount of Chla-N in the *Ov* plantlets, which was derived from ammonium assimilation, was not at the maximum level at the highest inorganic nitrogen concentration.

## Conclusions

We were able to distinguish the contribution of assimilated nitrate/ammonium to total inorganic nitrogen assimilation in plantlets *via* the bidirectional stable nitrogen isotope tracer technique. Although the concentration of nitrate was twice that of ammonium in all treatments, the utilization efficiency of nitrate was markedly lower than the utilization efficiency of ammonium for plantlets of both *Ov* and *Bn*. Ammonium was the primary source of nitrogen that was assimilated by *Ov* and *Bn* plantlets at a sufficient nitrogen supply. At the lowest inorganic nitrogen supply, the nitrogen demand of the *Ov* plantlets was mainly from the assimilation of nitrate. Moreover, considering the low inorganic nitrogen concentration in karst regions, where nitrate is more abundant than ammonium, plants with low inorganic nitrogen demands and strong ability to assimilate nitrate would be more adapted than would other plants to the soil conditions in karst regions. Hence, quantifying the utilization of nitrate and ammonium could provide a new way to reveal the differences in assimilating nitrate and ammonium among plant species at different inorganic nitrogen supply levels and contribute to optimizing the supply of inorganic nitrogen in culture media.

## Additional file

**Additional file 1: Table S1.** The leaf biomass of the *Ov* and *Bn* plantlets cultured under different inorganic nitrogen concentrations. Note: *Ov* *Orychophragmus violaceus*, *Bn* *Brassica napus*. The ratio of nitrate to ammonium within each inorganic nitrogen concentration was 2:1. Each value represents the mean ± SD (n = 3). Values signed with the same letter in each line are not significantly different by Tukey’s test (p > 0.05).

## Data Availability

All data generated or analyzed during this study are included in this published article and its Additional file 1: Table S1.
