# Supplementary material for: Differential contributions of NO3−/NH4+ to nitrogen use in response to a variable inorganic nitrogen supply in plantlets of two Brassicaceae species in vitro
Source: Plant Methods. 2019 Jul 31;15:86. doi: 10.1186/s13007-019-0473-1 (PMC6668107; doi:10.1186/s13007-019-0473-1)
Supplement: Supplementary file 1 — Additional file 1: Table S1. The leaf biomass of the Ov and Bn plantlets cultured under different inorganic nitrogen concentrations. Note: Ov Orychophragmus violaceus, Bn Brassica napus. The ratio of nitrate to ammonium within each inorganic nitrogen concentration was 2:1. Each value represents the mean ± SD (n = 3). Values signed with the same letter in each line are not significantly different by Tukey’s test (p > 0.05). [file 13007_2019_473_MOESM1_ESM.docx]

Table S1 The leaf biomass of the *Ov* and *Bn* plantlets cultured under different inorganic nitrogen concentrations

| Parameters | Plant species | Inorganic nitrogen concentration (mM) | | | |
| --- | --- | --- | --- | --- | --- |
|  |  | 20 | 40 | 60 | 80 |
| Leaf biomass (g) | *Ov* | 0.58±0.17a | 0.64±0.05a | 0.63±0.14a | 0.59±0.07a |
|  | *Bn* | 0.59±0.05c | 0.64±0.05bc | 0.80±0.07b | 1.01±0.08a |

Note: *Ov*-*Orychophragmus violaceus*, *Bn*-*Brassica napus*. The ratio of nitrate to ammonium within each inorganic nitrogen concentration was 2:1. Each value represents the mean ± SD (n=3). Values signed with the same letter in each line are not significantly different by Tukey’s test (p>0.05).
